# Downregulation of Sirt1 as aging change in advanced heart failure

**DOI:** 10.1186/1423-0127-21-57

**Published:** 2014-06-09

**Authors:** Tse-Min Lu, Jia-Yun Tsai, Yen-Chung Chen, Chun-Yang Huang, Hung-Lung Hsu, Chi-Feng Weng, Chun-Che Shih, Chiao-Po Hsu

**Affiliations:** 1 National Yang-Ming University, Institute of Clinical Medicine, School of Medicine, Taipei, Taiwan; 2 Division of Cardiology, Department of Internal Medicine, Taipei Veterans General Hospital, Taipei, Taiwan; 3 Division of Cardiovascular Surgery, Department of Surgery, Taipei Veterans General Hospital, 201, Section 2, Shih-Pai Road, Taipei 112, Taiwan; 4 Department of Pathology, National Yang-Ming University Hospital, Yi-Lan, Taiwan; 5 Department of Cardiovascular Surgery, Far Eastern Memorial Hospital, New Taipei, Taiwan

**Keywords:** Aging, Heart failure, Sirt1

## Abstract

**Background:**

In congestive heart failure the balance between cell death and cell survival in cardiomyocytes is compromised. Sirtuin 1 (Sirt1) activates cell survival machinery and has been shown to be protective against ischemia/reperfusion injury in murine heart. The role of Sirt1 in heart failure, especially in human hearts is not clear.

**Results:**

The expression of Sirt1 and other (associated) downstream molecules in human cardiomyocytes from patients with advanced heart failure was examined. Sirt1 was down-regulated (54.92% ± 7.80% in advanced heart failure samples compared with healthy control cardiomyocytes). The modulation of molecules involved in cardiomyocyte survival and death in advanced heart failure were also examined. The expression of Mn-superoxide dismutase and thioredoxin1, as well as an antiapoptotic molecule, Bcl-xL, were all significantly reduced in advanced heart failure cardiomyoctes (0.71 ± 0.02-fold, 0.61 ± 0.05-fold, and 0.53 ± 0.08-fold vs. control, respectively); whereas the expression of proapoptotic molecule Bax was significantly increased (1.62 ± 0.18-fold vs. control). Increased TUNEL-positive number of cardiomyocytes and oxidative stress, confirmed by 8-hydorxydeoxyguanosine staining, were associated with advanced heart failure. The AMPK-Nampt-Sirt1 axis also showed inhibition in advanced heart failure in addition to severely impaired AMPK activation. Increased p53 (acetyl form) and decreased FoxO1 translocation in the nucleus may be the mechanism of down-regulation of antioxidants and up-regulation of proapoptotic molecules due to low expression of Sirt1.

**Conclusion:**

In advanced heart failure, low Sirt1 expression, like aging change may be a significant contributing factor in the downregulation of antioxidants and upregulation of proapoptotic molecules through the p53, FoxO1, and oxidative stress pathways.

## Background

Despite recent progress, congestive heart failure remains a major cause of death in Western countries. The prevalence of congestive heart failure (CHF) increases with age [1]. Due to the increased average life expectancy over the past decade and the increased proportion of aged individuals, it is expected that CHF diagnoses will increase steeply in the near future.

There has been great progress towards understanding CHF pathogenesis and the implications of aging. Increasing evidence suggests that the progression of heart failure is determined by the imbalance between cell death promotion and cell survival/protective mechanisms [2]. In CHF or in an aging heart a predominant factor is the role of cell death [3].

Sirtuin 1 is a member of the Sirtuin family of class III histone deacetylases. Sirt1 is involved in gene silencing, longevity, cellular senescence, cell differentiation, and cell survival [4,5]. It has been shown that Sirt1 activity enhances the lifespan of several organisms, including yeast, C. elegans, and metazoan [6-8]. The protein expression of Sirt1 in the heart has been demonstrated to be the highest in the embryo with progressive reductions associated with aging [9]. These observations imply that Sirt1 may play an important role in the aging process, and with cardiomyocyte death and survival.

Sirt1 has been shown to inhibit apoptosis and to delay changes associated with aging in cardiomyocytes using a transgenic mouse as the model [10]. We have also found that Sirt1 expression was significantly reduced in hearts subjected to ischemic/reperfusion. Preconditioning before ischemic/reperfusion increased Sirt1 expression, and was associated with the upregulation of antioxidants and downregulation of proapoptotic molecules, partly through the activation of FoxO1 and reduced oxidative stress [11]. *In vitro*, endogenous Sirt1 has been found to play an essential role in mediating cardiomyocyte survival: overexpression of Sirt1 has provided protective effects for cardiomyocytes from apoptosis, whereas downregulation of Sirt1 may be associated with apoptotic cell death [12]. However, expression of Sirt1 in advanced heart failure models is not clear and remains controversial. Pillai et al. showed that Sirt1 is down-regulated in heart failure from human samples [13]: however, Alcendor et al. showed that Sirt1 is up-regulated after 4 weeks of pressure overload in mice, a condition used to represent the stage of heart failure [10].

In CHF, the ventricle or atrium wall tension is increased which subjects the cardiomyocytes to higher pressure and relative ischemia compared with normal healthy control hearts. It is implied that the molecular change following ischemia or ischemic/reperfusion in the mouse heart could be applied to the condition of CHF in human beings. Therefore, the goal of this study was to determine the expression of Sirt1 and the associated molecules involved in cell death or survival in a human model.

## Methods

### Collection of cardiac samples

This study was performed according to the guidelines of the Declaration of Helsinki. All procedures involved human tissue were approved by the institutional review boards of the Taipei Veterans General Hospital. Consent was obtained from patients or their families before tissue procurement. Myocardial samples over the posterior wall of the left atrium were collected from donors while being prepared for transplantation. Myocardial samples near the mitral annulus were obtained from patients at the time of therapeutic transplantation. Samples for biochemical analyses were stored at -80°C immediately after procurement. Selected samples for immunostaining were fixed in 4% paraformaldehyde in phosphate-buffered saline (pH 7.4), paraffin-embedded, and sectioned. C57BL/6JNarl mice were obtained from National Laboratory Animal Center of Taiwan, and the investigation conformed with the Guide for the Care and Use of Laboratory Animals published by the US National Institutes of Health.

### Cell cultures and materials

H9c2 cells (rat cardiomyoblasts), obtained from ATCC (Manassas, VA), and were cultured in DMEM supplemented with 10% fetal bovine serum under an atmosphere of 95% air/5% CO_2_ at 37°C. Compound C (Merck) was added to the medium for the times and at the doses indicated. The antibodies used included Sirt1, 8-OHdG goat polyclonal (Millipore), MnSOD (Upstate Biotechnology), Sirt1 rabbit polyclonal, Trx1, Troponin C mouse monoclonal (Santa Cruz), p53, Acetyl-p53 (Lys379), AMPKα and phospho-AMPKα(Thr172) (Cell Signaling), Nampt (Bethyl), Bcl-xL (Pharmingen), Bax (Abcam), FoxO1 rabbit monoclonal (Epitomics), and tubulin (Sigma).

### Immunoblot analysis

Heart samples were placed in lysis buffer (50 mmol/L Tris–HCl pH 7.4, 0.1% SDS, 1% NP40, 0.15 mol/L NaCl, 0.25% Na-deoxycholate, and 1 mmol/L EDTA supplemented with protease inhibitors). Densitometric analysis was performed using Scion Image software (Scion).

### Immunohistochemistry and immunofluorescence

The paraffin-embedded heart specimens, sectioned at 4-μm thickness, were deparaffinized and rehydrated in PBS. Pretreatments included microwave antigen retrieval in a 10 mM citrate buffer for 20 min. Immunohistochemical staining was performed using an immunocruz staining system (streptavidin-biotin peroxidase method), and sections were counterstained with hematoxylin. Immunofluorescence analysis was achieved using primary antibodies of Sirt1 (Santa Cruz), FoxO1 (Epitomics), acetyl-p53 (Cell Signaling), and/or Troponin C (Santa Cruz) after a blocking step with 3% BSA for 30 minutes. DyLight ™488-conjugated and/or ™594-conjugated affinipure IgG (Thermo Fisher Scientific) were used for secondary antibodies.

### Evaluation of apoptosis in tissue sections

Deoxyribonucleic acid (DNA) fragmentation was detected *in situ* using TUNEL Briefly, deparaffinized tissue sections were incubated with proteinase K, and DNA fragments were labeled with fluorescein-conjugated dUTP using TdT (Roche Molecular Biochemicals). Myocyte was identified by Troponin C antibody. Cell nuclei were counterstained with DAPI (blue).

### Statistics

All values were expressed as mean ± SEM. Statistical analyses between groups were done using a one-way ANOVA, and when F values were significant at a 95% confidence limit, differences among group means were evaluated using Fisher’s project least significant difference post-test procedure for group data. A P value less than 0.05 was considered significant.

## Results

Totally, the cardiac tissue samples were harvested from 10 donors (eight males, aged 37.5 ± 3.9 years) and 10 recipients of dilated cardiomyopathy (six males, aged 40.1 ± 3.7 years, *P* = 0.637). The cardiothoracic ratio of recipients was 67.65 ± 1.06%, measured by posteroanterior film of chest x ray.

### Sirt1 is down-regulated in advanced heart failure of human beings

**Figure 1:**
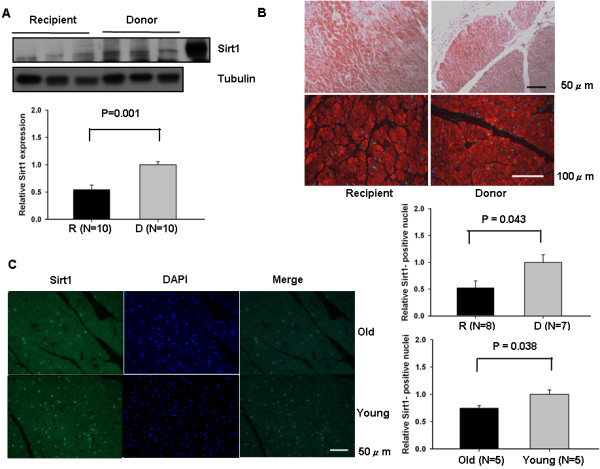
**Sirt1 is down-regulated in cardiomyocytes from patients with advanced heart failure.** Atrial myocardial homogenates were prepared from the recipient and donor heart sample. **(A)** Expression of Sirt1 and tubulin was evaluated by immunoblots. The level of donor heart is expressed as 1. H9c2 cells treated with Ad-Sirt1 was used as a positive control. **(B)** Atrial myocardial sections from the recipient and donor patients were subjected to hematoxylin-eosin staining (upper) and immunostaining (lower) with anti-troponin C antibody (red), anti-Sirt1 antibody (green), and DAPI (blue). The number of Sirt1 positive nuclei/total nuclei of donor heart is expressed as 1. **(C)** The ventricular myocardial sections of hearts from old (15 months) and young (3 months) mice were subjected to immunostaining with anti-Sirt1 antibody (green) and DAPI (blue). The number of Sirt1 positive nuclei/total nuclei of young heart is expressed as 1.

The expression of Sirt1 decreased to 54.92 ± 7.80% compared to donor hearts (Figure 1A). In advanced heart failure, atrial myocyte showed some hypertrophy and disarragement as expected (Hematoxylin-eosin staining in Figure 1B). Sirt1’s expression as observed using immunohistochemistry showed reduced nuclear expression (decreased to 52.74 ± 12.72% compared to donor) with advanced heart failure (Figure 1B). This result is similar to those observed in changes to the aging murine hearts (Figure 1C).

### MnSOD, Trx1, and apoptotic proteins are modulated in advanced heart failure

**Figure 2:**
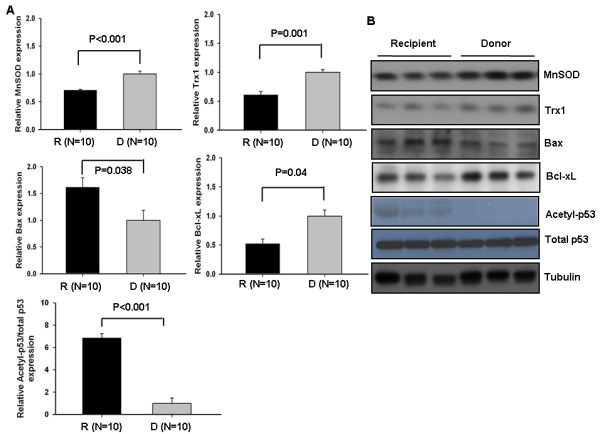
**MnSOD, Trx1, and apoptotic proteins are modulated in advanced heart failure.** Atrial myocardial homogenates were prepared from the recipient and donor heart sample. **(A)** Expression of MnSOD, Trx1, Bax, Bcl-xL, acetyl-p53, p53 (total) and tubulin was evaluated by immunoblots. The level of donor heart is expressed as 1. **(B)** Representative immunoblots.

The expression of downstream targets of Sirt1, such as Mn-superoxide dismutase (MnSOD) which is modulated by Sirt1 [14], Trx1, and the molecules associated with apoptotic signaling pathway were analyzed using immunoblot. The expressions of beneficial molecules, such as MnSOD, Trx1 and Bcl-xL (0.71 ± 0.02-fold, 0.61 ± 0.05-fold, and 0.53 ± 0.08-fold vs. donor, respectively) are all decreased in advanced heart failure (Figure 2A and B). The expression of Bax was significantly increased (1.62 ± 0.18-fold vs. donor) in advanced heart failure (Figure 2A and B).

### TUNEL-positive cardiomyocyte and oxidative stress are increased in advanced heart failure

**Figure 3:**
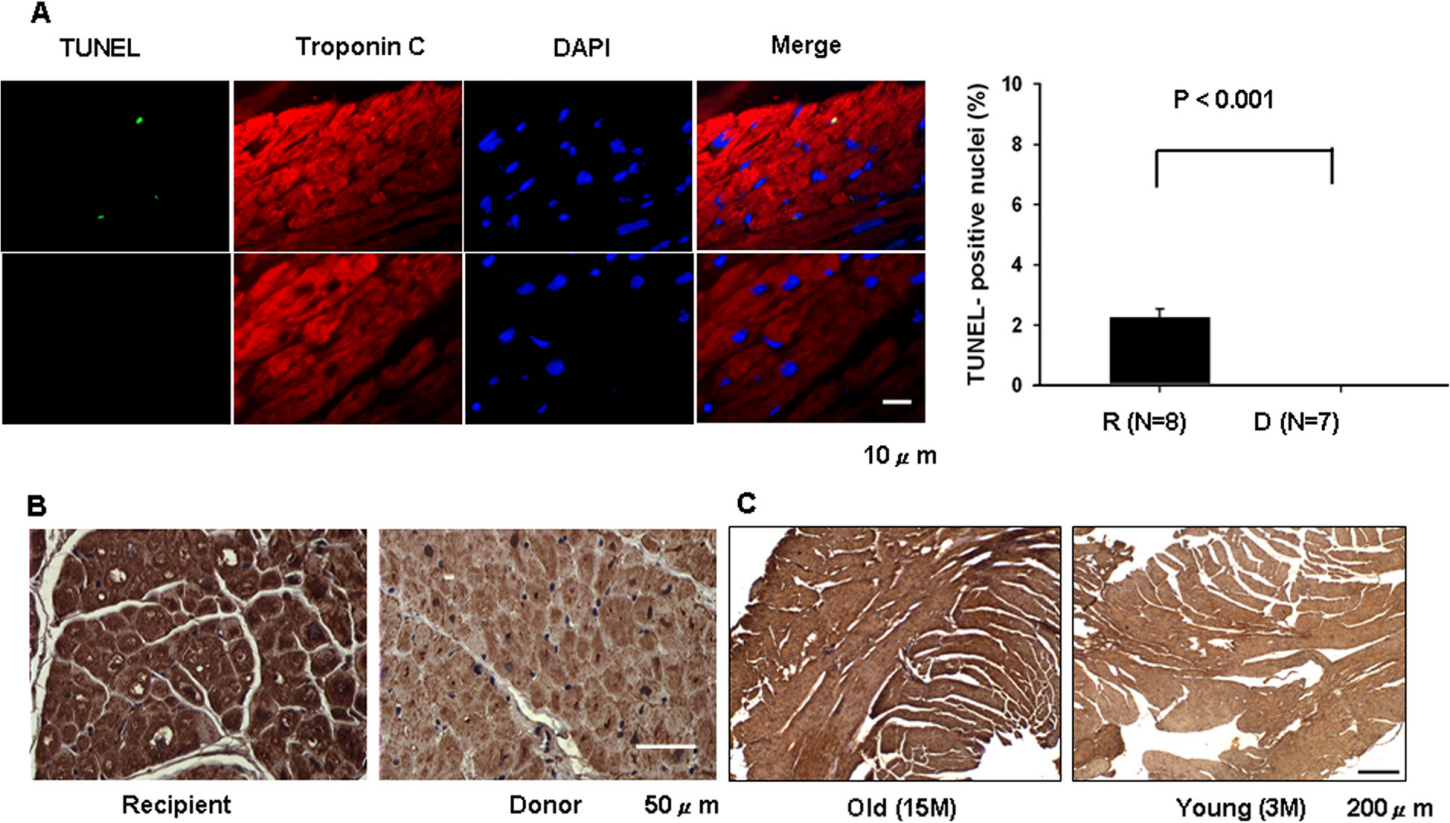
**Apoptosis and oxidative stress are aggravated in advanced heart failure.** Atrial myocardial sections from the recipient and donor patients were subjected to **(A)** TUNEL, troponin C and DAPI staining, the number of TUNEL-positive myocytes was expressed as a percentage of total nuclei detected by DAPI staining. and **(B)** 8-OHdG immunostaining. Representative images of the staining are shown. **(C)** The ventricular myocardial sections of hearts from old (15 months) and young (3 months) mice were subjected to immunostaining with 8-OHdG, and representative images of the staining are shown.

TUNEL staining for both recipient and donor hearts was performed to further investigate changes associated with aging. The number of TUNEL positive cardiomyocytes in the heart failure group was increased significantly compared with normal donor control cardiomyocytes (Figure 3A). Due to the lower expression of Trx1 in advanced heart failure, differences in oxidative stress of the myocardium were further examined. 8-hydroxydeoxyguanosine (8-OHdG), a critical marker of oxidative damage of genomic DNA and the aging process [15,16], was characterized using immunohistochemistry. Results showed increased oxidative stress in both nucleus and cytosol of cardiomyocytes in advanced heart failure, similar to the changes observed in aged cardiomyocytes (Figure 3B and C).

### The activity of the AMPK-Nampt-Sirt1 axis is decreased in the advanced heart failure

**Figure 4:**
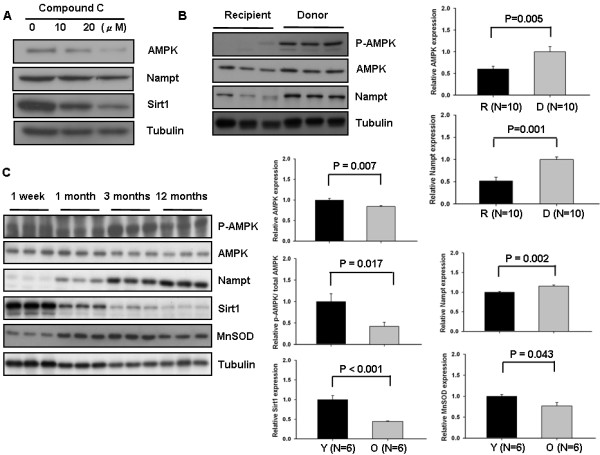
**The activity of the AMPK-Nampt-Sirt1 axis is decreased in the advanced heart failure. (A)** H9c2 cells were treated with compound C at indicated concentrations and cultured for 24 hours. Cell lysates were subjected to immunoblot analyses with anti-AMPK, anti-Nampt, anti-Sirt1 and anti-tubulin. The results shown are one representative of four independent experiments. **(B)** Atrial myocardial homogenates were prepared from the heart recipient and donor patients, respectively. Expression of phospho-AMPK(Thr172), AMPK, Nampt, and tubulin was evaluated by immunoblots. The level of donor heart is expressed as 1. Representative immunoblots were shown. **(C)** Heart homogenates were prepared from C57/B6 mice aged 1 week, 1 month, 3 months, and 12 months. Expression of phospho-AMPK(Thr172), AMPK, Nampt, Sirt1, MnSOD and tubulin was evaluated by immunoblots, and the level of phospho-AMPK/total AMPK, Nampt, Sirt1, and MnSOD was compared between 3-month (expressed as 1) and 12-month mice.

Since both AMPK and Nampt are the upstream molecules of the functional signaling pathway of the AMPK-Nampt-Sirt1 axis [17], we examined their expression in advanced heart failure. Whether the axis of AMPK-Nampt-Sirt1 was functional in the heart was determined under the treatment of compound C, an AMPK inhibitor. The expression of both Nampt and Sirt1 was decreased in H9c2 cells, which implied that the axis may work in the heart (Figure 4A). The expression of both AMPK and Nampt was also decreased (0.60 ± 0.06-fold and 0.52 ± 0.08-fold vs. donor, respectively) in advanced heart failure as Sirt1’s expression (Figure 4B). Furthermore, the expression of phospho-AMPK was severely inhibited in advanced heart failure (Figure 4B). In order to confirm these results, hearts from an aging C57/B6 mouse was examined. The expression of AMPK, Sirt1 and MnSOD was decreased in the old mice (aged 12 months) compared with younger mice (3 months), but the expression of Nampt was increased. The expression of phospho-AMPK/total AMPK was also checked and showed significantly decreased (or inhibited) in the old mice (aged 12 months) (Figure 4C).

### Translocation of Sirt1 and regulation of FoxO1 and p53 in advanced heart failure

**Figure 5:**
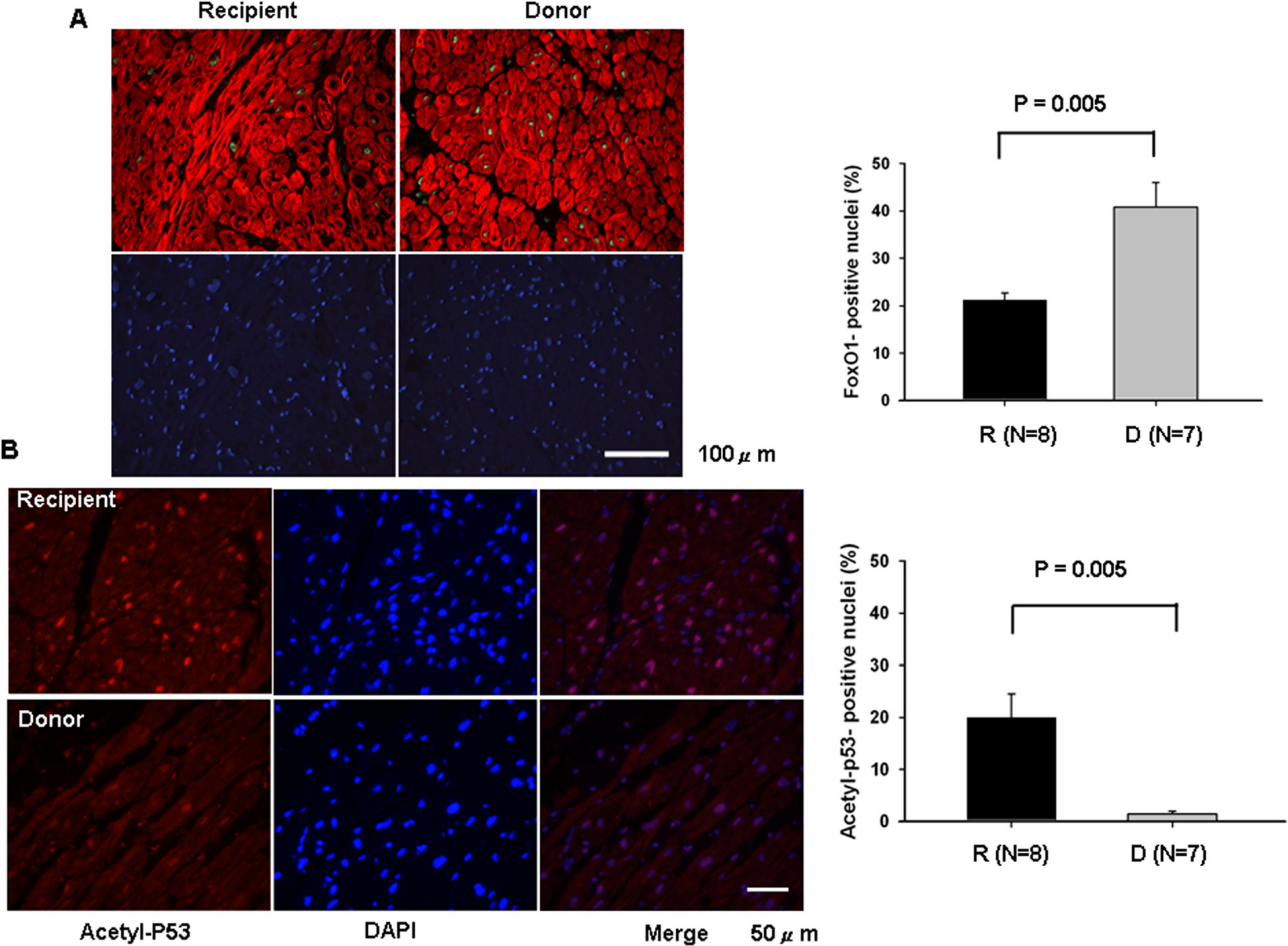
**Sirt1 modulates p53 and nuclear localization of FoxO1 in advanced heart failure. (A)** Atrial myocardial sections from the heart recipient and donor patients, respectively were subjected to immunostaining with anti-FoxO1 antibody (green), anti-troponin C antibody (red), and DAPI (blue). The number of FoxO1-positive nuclei was expressed as a percentage of total nuclei. **(B)** Atrial myocardial sections from the heart recipient and donor patients were subjected to immunostaining with anti-acetyl p53 antibody (red) and DAPI (blue). The number of acetyl-p53 positive nuclei was expressed as a percentage of total nuclei detected by DAPI staining.

Due to lower expression of Sirt1 and MnSOD in heart failure, translocation of transcriptional factor FoxO1, which could modulate the expression of MnSOD [18], was examined, Immunostaining revealed decreased FoxO1 in the nucleus in advanced heart failure cardiomyocytes (Figure 5A). Furthermore, because of the increased expression of Bax, a downstream effector that is positively regulated by p53 in heart failure [19], we examined acetylated p53, which was significantly increased in advanced heart failure (Figure 5B).

## Discussion

This is a cross-sectional study designed to investigate the expression of Sirt1 and its associated molecules in the left atrium from the end-stage heart failure patients, and compared those from even younger donors. Simultaneously, these changes in heart failure are found to be similar to those observed in aging mice. Both of our study and Sakamoto et al’s, [9] showed that the expression of Sirt1 in the heart is decreased with age. The gap in age, in spite of statistical insignificance, may have some minor effect on the Sirt1’s expression. Hence, we checked whether the expression of Sirt1 is affected by aging effect in our sample (Additional file 1: Figure S1). It showed that it could be neglected, and the observations in this study are mostly related to progressive genetic remodeling of the failing heart.

It is widely agreed that Sirt1 plays a cardioprotective role in many aspects. There have been various studies conducted on *in vivo* heart models [11,20], *ex vivo* Langendorff model [11,20,21], or cultured cardiomyocytes [10,12,22], which all suggested that Sirt1 serves a protective role against cardiovascular stresses through the regulation of apoptosis inhibition, aging retardation, and oxidative stress alleviation [6,8,10].

We currently demonstrate that Sirt1 is down-regulated in the myocardium in patients with advanced heart failure, The result, as was supposed, is very similar to our previous report [11], and consistent with the report of human samples by Pillai et al. [13]. Alcendor et al. [10], showed that Sirt1 is up-regulated (8.8-fold of protein expression) after 4 weeks of pressure overload in mice, a condition used to represent the stage of heart failure, however, they did not check the cardiac function of these mice, nor the activity of Sirt1. Increase of Sirt1 expression in the heart was associated with cardiac hypertrophy [10,23], however, the constitutive high level of Sirt1 may be harmful to the heart itself and reduce cardiac function, proved by transgenic model [10,24]. It also points out that the expression level of Sirt1 is important, too much is as bad as too little. In our study, we found that Sirt1 is obvious decreased in the heart of 12 or15 month mice, and a significant decline in left ventricular systolic function was not noted until 18 months of age in the C57BL/6J mice [25], the same background of mouse as our study. Taken above findings altogether, it might be assumed that Sirt1 may be highly up-regulated to protect the cardiomyocytes in the early stage of heart failure (compensated stage) associated with pressure-overload, which could be explained the finding of elevation of Sirt1’s expression in dog by Alcendor et al. [12], however, the sustained too high level of Sirt1 is not beneficial, but detrimental to the cardiomyocytes through unclear mechanisms and the expression of Sirt1 may be progressively decreased accompanying the injury of cardiomyocytes in the long run (decompensated stage), and contributes partly to heart failure finally. More experiments are necessary to investigate the hypothesis. Furthermore, the reason for the discrepancy about higher expression of Sirt1 in old monkeys by Alcendor et al. [10] remains to be elucidated, and whether any stress impacted on these old monkeys or not should be explored.

We have previously reported that Sirt1 can stimulate the expression of MnSOD in cultured cardiomyocytes, for which FoxO1 has been shown to play an essential role [11], and in response to serum starvation *in vitro*, the deacetylation of p53 by Sirt1 inhibits cardiac myocytes apoptosis [12]. We currently report that in advanced heart failure, both the FoxO1 and deacetylation of p53 were significantly reduced in the nuclei of cardiomyocytes. These may be the mechanisms of reduced MnSOD, Bcl-xL, and increased Bax, apoptosis, and oxidative stress with the downregulation of Sirt1 expression.

Regardless of the stressor, downregulation of Sirt1 in the heart was associated with loss of cardioprotection in the literatures, such as our result that low expression of Sirt1 in advanced heart failure may lead to additional irreversible heart injury via downstream effectors. However, further studies are necessary to fully elucidate the mechanism of how Sirt1 down-regulated, especially the relationship between Sirt1 and mechanical or neurohormonal stimulus of the failing heart before we target it.

Sirtuin activity is regulated by NAD^+^ biosynthetic pathways, in which Nampt plays a critical role as a regulator for NAD^+^ synthesis in cardiomyocytes [26]. Fulco et al. showed that the axis of AMPK-Nampt-Sirt1 was a functional pathway to sense and react to nutrient availability in skeletal muscle cells [17]. We also examine it in our samples and find the expression of Nampt is inconsistent in aged murine hearts and advanced heart failure. In aged heart, the expression of Nampt is increased, which may be secondary to compensate for the decrease of Sirt1. Moreover, in the mouse sample, the expression of phospho-AMPK and MnSOD is not synchronous to that of Sirt1 in the early age. The activation of phospho-AMPK in the early neonatal life may be likely associated with starvation (fasting) after birth, and it may imply that the regulation of these molecules is more complicated than our mention.

Our result suggests that the AMPK-Nampt-Sirt1 pathway may be disordered in heart failure and the mechanism of aging and heart failure is similar but not exactly the same. It also implicates that multiple molecular pathways involved in determining aging process. Recently, Ma et al. [27] also showed that ischemic AMPK activation was impaired in aged murine hearts, and Reznick et al. [28] disclosed that aging-associated reductions in AMPK activity may be an important contributing factor in reduced mitochondrial function and dysregulated intracellular lipid metabolism. We also report similar results of AMPK change in aged murine hearts. Per results provided by Fulco et al. glucose restriction (or nutrient shortage) functions to keep skeletal myoblasts from differentiation and hold them in the younger (undifferentiated) stage through activated AMPK. Although the exact molecular mechanisms through which nutrients influence various cell signaling/modulators of lifespan remain a largely unresolved issue. However, the above findings showed that the AMPK and/or activated AMPK, and the modulated Sirt1, may be regarded as important regulators or markers of aging, even the differentiation of cardiomyocytes. Upregulation of the functioning pathway can be an alternative method apart from increasing Sirt1’s activity or expression, as a cardioprotective intervention. The metabolism of nutrition in the myocardium will change substantially during advanced heart failure, however, further experiments are necessary to elucidate the role of Sirt1 on the metabolic remodeling of the failing heart.

### Clinical relevance

Downregulation of Sirt1 may have important meaning in the advanced heart failure, though cause and effect relationship is not yet established in our observation. Sirt1 agonists, such as Resveratrol, has been shown to protect the heart from ischemia/reperfusion [29]. Recently, Telmisartan, an angiotensin receptor blocker used in the management of hypertension or heart failure, was found to activate the AMPK-Sirt1 pathway in skeletal muscle, and ameliorate insulin sensitivity of obese db/db mice [30]. These Sirt1 agonists may prevent changes associated with cardiac aging, and targeting Sirt1 may become a promising modality to protect the heart from aging or heart failure.

### Limitation

There are several limitations in the study. One is because of impossibility to obtain the ventricular sample from the donor heart, we only used left atrium samples instead of left ventricle. Even though, we can not get the similar region from left atrium between donor and recipient heart while heart transplantation. Another is that the cause of heart failure is dilated cardiomyopathy in our study, younger than congestive heart failure due to ischemic heart disease, hypertension, or valvular heart disease. The observations may not be applied completely to other causes of congestive heart failure. Further investigations may be necessary to explore the difference.

## Conclusions

In conclusion, in advanced heart failure, the changes associated with aging may be accelerated and the cardioprotective effect of Sirt1 is reduced due to the lower expression level and changes to downstream effector molecules. In addition, the reduced overall expression of AMPK-Nampt-Sirt1 may partly explain the molecular basis of this mechanism.

## Competing interests

The authors declare that they have no competing interests.

## Authors’ contributions

T-ML and J-YT carried out the molecular studies. J-YT drafted the manuscript. Y-CC carried out the immunostainings. C-YH performed the statistical analysis, H-LH and C-FW perfromed the sample harvesting and preparation. T-ML participated in its design. C-CS and C-PH conceived of the study, and participated in its design and coordination and helped to draft the manuscript. All authors read and approved the final manuscript.

## Supplementary Material

Additional file 1: Figure S1 The regression analysis of relative expression of Sirt1 and age of patient in our sample. The coefficient of determination (R-Square) of both donor and recipient sample is 0. Click here for file
