# Supplementary figures and images for: Downregulation of Sirt1 as aging change in advanced heart failure
Source: J Biomed Sci. 2014 Jun 9;21(1):57. doi: 10.1186/1423-0127-21-57 (PMC4113120; doi:10.1186/1423-0127-21-57)

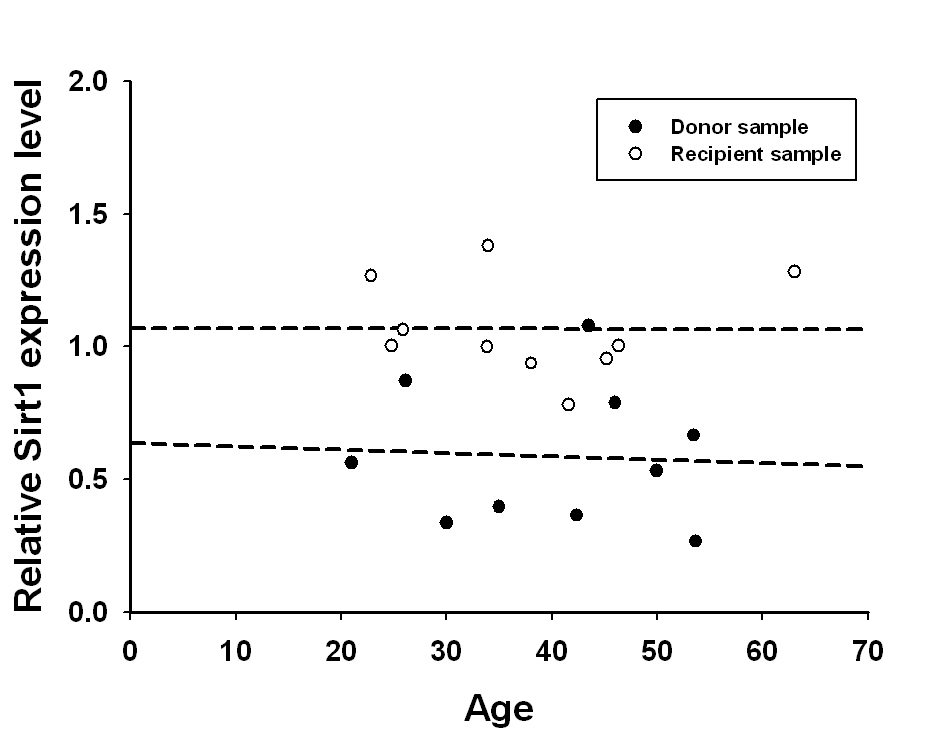

Supplement: Additional file 1: Figure S1 — The regression analysis of relative expression of Sirt1 and age of patient in our sample. The coefficient of determination (R-Square) of both donor and recipient sample is 0. [file 1423-0127-21-57-S1.jpeg]
